# Epistatic Interactions in NS5A of Hepatitis C Virus Suggest Drug Resistance Mechanisms

**DOI:** 10.3390/genes9070343

**Published:** 2018-07-06

**Authors:** Elena Knops, Saleta Sierra, Prabhav Kalaghatgi, Eva Heger, Rolf Kaiser, Olga V. Kalinina

**Affiliations:** 1 Institute of Virology, University of Cologne, 50935 Cologne, Germany; elena.knops@uk-koeln.de (E.K.); saleta.sierra-aragon@uk-koeln.de (S.S.); eva.heger@uk-koeln.de (E.H.); rolf.kaiser@uk-koeln.de (R.K.); 2 German Center for Infection Research (DZIF)—Cologne-Bonn Partner Site, 50935 Cologne, Germany; 3 Department of Computational Biology and Applied Algorithmics, Max Planck Institute for Informatics, 66123 Saarbrücken, Germany; prabhavk@mpi-inf.mpg.de; 4 German Center for Infection Research (DZIF)—Saarbrücken Partner Site, 66123 Saarbrücken, Germany

**Keywords:** hepatitis C virus, NS5A, drug resistance, epistasis, protein structure

## Abstract

Hepatitis C virus (HCV) causes a major health burden and can be effectively treated by direct-acting antivirals (DAAs). The non-structural protein 5A (NS5A), which plays a role in the viral genome replication, is one of the DAAs’ targets. Resistance-associated viruses (RAVs) harbouring NS5A resistance-associated mutations (RAMs) have been described at baseline and after therapy failure. A mutation from glutamine to arginine at position 30 (Q30R) is a characteristic RAM for the HCV sub/genotype (GT) 1a, but arginine corresponds to the wild type in the GT-1b; still, GT-1b strains are susceptible to NS5A-inhibitors. In this study, we show that GT-1b strains with R30Q often display other specific NS5A substitutions, particularly in positions 24 and 34. We demonstrate that in GT-1b secondary substitutions usually happen after initial R30Q development in the phylogeny, and that the chemical properties of the corresponding amino acids serve to restore the positive charge in this region, acting as compensatory mutations. These findings may have implications for RAVs treatment.

## 1. Introduction

Hepatitis C virus (HCV) is a major world health threat. In 2015, it caused 1.34 million deaths; 71 million people live with the infection worldwide. It causes chronic hepatitis, liver cirrhosis, and liver cancer [1]. Recently, effective treatment with direct-acting antivirals (DAAs) has become available [2]. These drugs specifically inhibit one of the viral proteins NS3/protease, non-structural protein 5A (NS5A) or 5B (NS5B). Worldwide eradication of HCV infection is not expected soon, as only 0.7% of the infected population has gained access to DAAs so far [3]. HCV is classified into seven genotypes and 67 sub/genotypes (GTs) [2]. DAA susceptibility varies along the different viral GTs, and therefore some DAAs are approved only for certain subtypes [2]. DAA susceptibility varies among the different viral GTs, and therefore some DAAs are approved only for certain subtypes [4].

The NS5A is a target for therapy of HCV, yet the precise function(s) of this protein is/are not elucidated. This protein is known to be membrane-bound and plays an essential role in HCV replication [5]. It has many known and putative cellular interaction partners, and many specific inhibitors of it have been identified [6]. NS5A-directed inhibitors are extremely potent, inhibiting viral replication in picomolar concentrations [7,8]. For these drugs, not only is there different susceptibility among GTs, but also there is evidence of different resistance-associated-mutations (RAMs) development [7,9]. Importantly, NS5A RAMs have been described both before treatment (also called baseline) as well as after failure of NS5A inhibitors-containing therapies [10,11,12,13,14,15]. According to the current recommendations of the European Association for the Study of the Liver (EASL), resistance testing for NS5A inhibitors is subject to the availability of reliable tests, and the list of potential NS5A RAMs include several mutations in positions 28, 30, 31, 58, and 93 for GT-1a viruses and the mutation Y83H for the GT-3 viruses [10]. No RAMs for GT-1b are listed in the guidelines. However, there is evidence that certain mutations in positions 28, 31, 32, 92, and 93 can also confer resistance to NS5A inhibitors in GT-1b viruses [16,17,18,19,20,21,22,23]. Due to differences in the consensus sequences among GTs, the specific amino acid substitutions corresponding to RAMs differ across genotypes (or even across subtypes), while their positions remain predominantly the same. Intriguingly, the consensus residue in position 30 for the GT-1b viruses is an arginine (R), in contrast to the GT-1a, where it is a glutamine (Q). Q30R is known to confer resistance against multiple NS5A inhibitors for GT-1a viruses [16,17,23,24,25]. The substitution to a chemically similar lysine (K) has been observed to confer resistance both for GT-1a viruses [26] and for GT-3 viruses [11].

**Figure 1:**
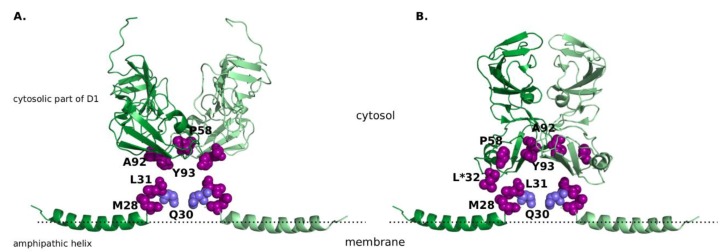
The non-structural protein 5A (NS5A) structure with R30 highlighted in light-blue and other resistance-associated mutations highlighted in purple, all shown in the sphere mode. (**A**) “Claw-like” conformation of NS5A dimer. Amphipathic helix is taken from the nuclear magnetic resonance (NMR) structure (Protein Data Bank, PDB ID 1R7E [29]), cytosolic subdomain of domain 1 from the X-ray crystal structure (PDB ID 1ZH1 [28]). (**B**) “Back-to-back” conformation of NS5A dimer. Amphipathic helix is taken from the NMR structure (PDB ID 1R7E [29]), cytosolic subdomain of domain 1 from the X-ray crystal structure (PDB ID 3FQM [28]). It must be noted that positing of the amphipathic helix is not based on experimental data, and solely aims to display all resistance-associated mutations in close proximity to each other. Amino acid identities are displayed as they are given in the experimental structures, the corresponding amino acid identities for sub/genotype GT-1a and GT-1b are listed in Table 1. Position 32, in which amino acid identity differs from the naturally occurring amino acids, is marked with an asterisk.

NS5A is a phosphoprotein that comprises three domains, of which only the most *N*-terminal domain D1 has an experimentally resolved three-dimensional structure. It consists of an amphipathic alpha-helix at its *N*-terminal end that is anchored in the membrane, and cytosolic subdomain of D1 that predominantly contains beta structures, joined by a flexible linker region. D1 tends to form dimeric and higher-order assemblies [27,28]. The full-length structure of the D1 has yet to be determined, but multiple X-ray and nuclear magnetic resonance (NMR) structures exist for its individual parts: the amphipathic alpha-helix [29], and the cytosolic subdomain [27,28]. However, the mutual orientation of the amphipathic helix and cytosolic part, as well as of the two amphipathic helices in the context of a dimer, is not known. The NS5A dimer assumes at least two distinct conformations (reviewed in [30]). One of these conformations is thought to bind RNA in a groove facing away from the membrane between cytosolic subdomains of D1 of the two subunits, so-called “claw-like conformation” ([27], Figure 1A). In the other conformation, this groove is buried (“back-to-back” conformation ([28], Figure 1B). However, a binding site between the two cytosolic subdomains of D1 and the corresponding linker to the amphipathic helices can be modelled at the theoretical membrane-interacting surface of either dimer complex [30]. It has been suggested that binding of DAAs to this binding site reduces the affinity of NS5A to RNA thus stabilizing one of the two conformations [31], directing NS5A from the endoplasmic reticulum to lipid droplets, and thus inhibiting the formation of new replication complexes [7,32]. The above-mentioned RAMs are shared between the amphipathic alpha-helix and the cytosolic subdomains of D1 (Figure 1, Table 1). 

Several modelling attempts have been undertaken to suggest models of the full-length *N*-terminal domain of NS5A containing the amphipathic α-helix, the linker region and cytosolic subdomain in the context of DAA binding [31,33,34,35,36,37]. In some cases, the DAA inhibitor was docked directly to an isolated structure of D1 dimer [36]. There is no consensus on the orientation of the amphipathic α-helix in these models: in some cases it can be modelled parallel to each other in the two monomers with its *N*-terminus pointing to the centre of the dimer [37], sometimes away from the dimer [33,34], and in other analysis kinked and pointing sideways [33]. In most cases the inhibitor can be modelled to bind symmetrically [6,31,33,34,35,36]. Nevertheless, asymmetric binding has also been proposed [37]. Though these modelling studies partly explain the RAMs observed in NS5A in different genotype contexts, there is no direct evidence for the mode of binding of DAAs to this cleft.

In this study, we present an analysis of epistatic interactions in NS5A, both in the context of evolution of strains belonging to the GT-1b, and in the context of the three-dimensional structure of the NS5A protein. Specifically, we were interested to analyse whether the substitution of the consensus arginine to a glutamine in the GT-1b viruses is observed frequently enough to be considered a common polymorphism in this genotype, and whether it interacts epistatically with other mutations in the NS5A protein. We observed that R30Q is a frequent variant in the general population of the GT-1b sequences. We discovered several other mutations that are frequently developed following the R30Q change and can arise independently in evolution. Finally, we discuss possible implications for resistance to DAAs and novel therapy options. Our new data may shed light on whether any of the previously proposed models correctly represents DAA binding to NS5A in vivo. In addition, this study identifies new amino acid substitutions that may also have a role in viral resistance to NS5A inhibitors.

## 2. Materials and Methods 

5465 HCV NS5A sequences were downloaded from the HCV sequence database [38], retaining the original genotype annotations. They were aligned with MACSE (Multiple alignment of coding sequences) [39] to account for frame-shifts and stop codons. For phylogeny reconstruction, 669 duplicate sequences were removed and the remaining 4799 unique sequences were analyzed. Sequence evolution was modelled using a general time reversible model with T-distributed rate variation among sites (GTR + Gamma) model of substitution, and a maximum likelihood phylogenetic tree was inferred using RA×ML (v 8.2.8) [40]. The maximum-likelihood tree was rooted such that one of the branches leading from the root was monophyletic in GT-1b and contained 2558 GT-1b sequences, and the other branch leading from the root contained 2182 GT-1a sequences and 59 GT-1b sequences. The 59 sequences were annotated as GT-1b but located within the GT-1a branch were considered to be wrongly annotated. They correspond to 73 sequences in the original data not filtered for identical sequences, and these 73 were removed from this study. The maximum likelihood phylogenetic tree **T** was pruned by removing the wrongly annotated sequences, and was rooted by placing the root between the GT-1a and GT-1b monophyletic branches. Ancestral states were reconstructed under a GTR + Gamma model of substitution using RA×ML (v 8.2.8). The final full set of sequences (Supplementary File 1), the reconstructed phylogenetic tree (Supplementary File 2) and sequences in the internal nodes (Supplementary File 3) are available in the Supplementary Materials.

For statistical analysis, we used 5394 sequences that remained in the original data after removing 73 wrongly annotated sequences, 2617 of them annotated as GT-1a and 2777 annotated as GT-1b (Supplementary File 1). Numbering of the residues was done by alignment with H77 (NCBI Reference Sequence accession number NP_751927.1). 2777 GT-1b sequences were used for the subsequent modelling analysis. They were split into two classes based on the identity of the amino acid in position 30: those with arginine in this position are called here typical (since this is the consensus amino acid in this position for the GT-1b), and those with a glutamine are called atypical. In all other positions of the atypical sequences the most frequent amino acid coincides with the consensus amino acid for GT-1b. However, the second most frequent residue sometimes corresponds to the consensus for the GT-1a. These positions were selected, and the overrepresentation of the residue characteristic for the GT-1a among the atypical sequences compared to the typical sequences was tested with Fisher’s exact test, the Bonferroni correction was applied to account for multiple testing (Table 2). For each pair of the selected positions *i* and *j*, the correlation of substitutions within the set of atypical sequences was calculated as odds ratios: *f*(*i*_1a, *j*_1a)/(*f*(*i*_1a) * *f*(*j*_1a)), where *f*(*i*_1a, *j*_1a) is the frequency to observe the amino acid residues characteristic for the GT-1a both in positions *i* and *j* simultaneously, *f*(*i*_1a) and *f*(*j*_1a) are the frequencies to observe such a residue in each of the positions separately. The significance of the correlation was tested with the Fisher’s exact test; again, the Bonferroni correction was applied to account for multiple testing (Table S1).

The order of the mutations was examined using the phylogenetic tree: for each terminal node corresponding to an atypical sequence (hence with a glutamine in position 30), a path to the nearest internal node with an arginine in position 30 was reconstructed, and the identity of amino acids in all selected positions with significantly overrepresented second most frequent residue corresponding to GT-1a was monitored along this path to detect whether the corresponding mutations happened before or after the switch of arginine to glutamine, or did not happen in the particular sequence at all. In cases when such mutations happened after the switch, they were called compensating (Table S2). To test whether the same pattern of mutations is likely to arise by chance in this tree, a random model was constructed as follows: a set of random internal nodes of the same depth as the nodes corresponding to the observed arginine to glutamine switches was selected 10,000 times, for each node the corresponding number of terminal nodes was randomly selected from the induced subtree (a single switch node may correspond to multiple terminal atypical sequences), and the number of residues characteristic of GT-1a was calculated in these terminal nodes for each selected position. This distribution was used as the background to calculate phylogeny-aware statistical significance of the mutations (Table 2).

The three-dimensional protein structures were obtained from the Protein Data Bank (http://www.wwpdb.org/), and the following structures were used: 1ZH1 and 3FQM for the cytosolic part of D1, and 1R7E for the amphipathic alpha-helix. Additionally, all identified compensating residues were then mapped into the three-dimensional structure of the NS5A dimer in complex with the Daclatasvir inhibitor, corresponding to the mode-II proposed by Nettles et al. [37]. The potential compensating residues corresponding to the key correlated mutations (Table 2, bold and underlined), as well as resistance-associated residues were highlighted and visualised in space-filling mode to evaluate their potential interactions using PyMol (version 2.0, Schrödinger, LLC). No additional modification or minimisation of the structure was performed. Additionally, the potential role of the residues corresponding to the key correlated mutations was estimated from theoretical images and discussions from other modelling studies [31,33,34,35,36], for which no coordinates for the models were available.

To identify group-specific positions, we applied multi-Harmony [41] and SDPfox [42] with default parameters. We used CD-HIT [43] with an identity cutoff of 98% to reduce the number of sequences to a value below 2000 in order to conform to the requirements of the SDP fox website. We also used the filtered set for GT-1a vs. GT-1b analysis with multi-Harmony, because the server did not return any results for the full set. We use SH Z-score cut-off of less than 5 to select the resulting set of groups-specific positions.

Three hundred sixty-four additional NS5A sequences from patients from the P.E.P.S.I. cohort [44] were also analysed. All patients enrolled in the P.E.P.S.I. Study gave their written consent allowing the use of their samples for scientific purposes. The P.E.P.S.I. Study has been approved by the ethics committee of the Medical Council North Rhine (Ärztekammer Nordrhein, Düsseldorf, Germany), No. 2012048. Resistance testing was performed using specific primers for the NS3, NS5A and NS5B regions, next generation sequencing (NGS), and the tool geno2pheno_[HCV]_ [45].

## 3. Results

### 3.1. Identification of Mutations Associated with GT-1b R30Q

**Figure 2:**
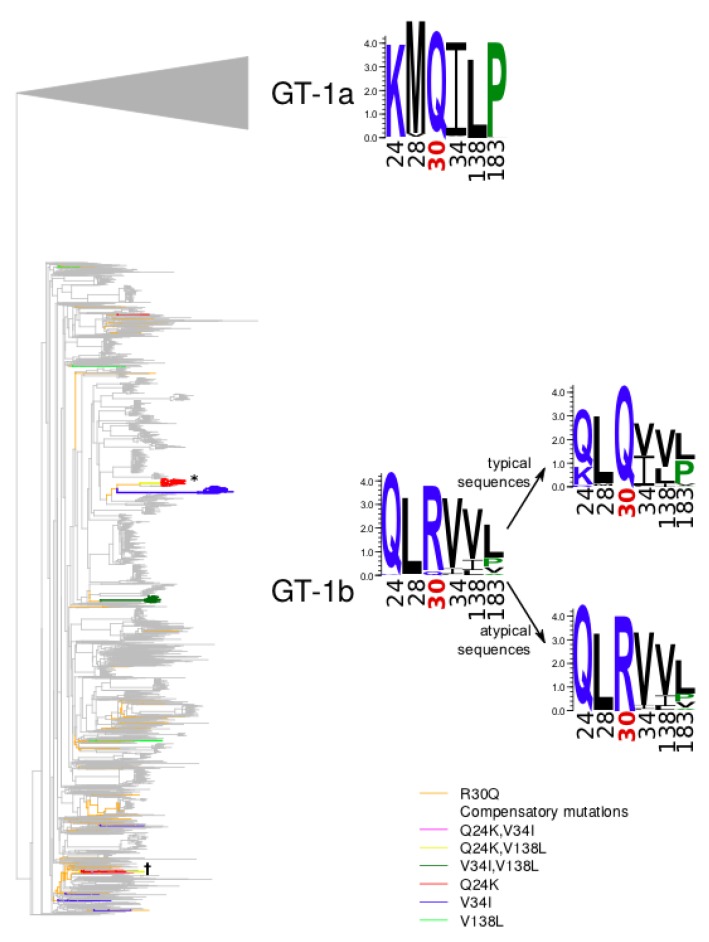
Phylogenetic tree of the GT-1b sequences of NS5A. Branches leading from reconstructed R30Q mutation events to atypical sequences are marked in orange. Branches carrying other compensatory mutations are marked in red (Q24K), blue (V34I), green (V138L), pink (Q24K, V34I), yellow (Q24K, V138L), dark green (V34I, V138L).

2617 (GT-1a) and 2848 (GT-1b) HCV NS5A protein sequences from the HCV sequence database were used for the initial phylogenetic analysis. None of the 2617 GT-1a sequences displayed the Q30R mutation, and therefore none of them could be used for the in silico analysis. 71 sequences were annotated as GT-1b and were located inside the branch corresponding to GT-1a. We have additionally genotyped these sequences with geno2pheno_[HCV]_ using the set of reference sequences from Smith et al. [4], and found closest hits to GT-1a clade II sequences (93.2 to 94.44% sequence identity), and thus considered these sequences to be wrongly annotated as GT-1b and discarded them. The remaining 2777 GT-1b sequences were used for the subsequent modelling analysis, while 2636 wild-type (wt) sequences with an arginine at position 30 (R30) were used as the background typical set. The remaining 141/2777 (5%) sequences contained atypical glutamine residues at position 30 (R30Q) and constituted the atypical set. The sequences from the atypical set did not cluster in a single branch of the phylogenetic tree, indicating that the R30Q mutations occurred several times in independent evolutionary events in the GT-1b (Figure 2). In some of these sequences, additional mutations characteristic of GT-1a were found. We identified 15 positions, in which the second most frequent residue is the consensus amino acid residue of GT-1a, and statistical analysis suggested that some of such mutations are overrepresented in the atypical set (Table 2). Particularly prominent was the association of R30Q with the mutation Q24K. The residue 24 is located in the same flexible linker loop connecting the amphipathic *N*-terminal helix of NS5A to the structured domain 1 as the residue in the position 30. The mutation Q24K introduces a positively charged lysine and is complementary to the R30Q mutations in terms of physical properties of the residues.

Other residues that were significantly associated to the R30Q in GT-1b samples included the mutations L28M, V34I, Q54H, V138L, and L183P (with a Bonferroni-corrected *p*-value threshold of 0.05/15 = 0.00333333, Figure 1). All these mutations, except Q54H, V138L and L183P, are located in the same linker region between the amphipathic helix and the domain 1. V138 and L183 are located in the structured domain 1 in a loop, and there is no immediate communication between these residues and the inhibitor-bonding pocket. Whereas Q24K/R restores the positive charge in this region, the other mutations do not significantly change the properties of the amino acid residues. The only exception is L183P, a hydrophobic residue that typically perturbs the conformation of the backbone. However, residue 183 is the last one of a β-strand at the surface of the domain D1, so such perturbation is unlikely to cause any major functional effects. Q54H is one of the residues involved in the hydrogen bond network of the model by Barakat and colleagues [33] and indeed the substitution for histidine (H) provides an additional hydrogen bond that was lost upon the mutation R30Q, and the histidine may assume the same positive charge as the arginine. However, this mutation is not supported by our phylogenetic test (see Section 2 for details), so we did not consider it for further analysis.

To test whether the same set of mutations is recurring in the same set of atypical sequences, we calculated the pairwise correlation of all selected residues, now excluding position 30 from the consideration (Table S1). After applying the Bonferroni correction, we observed that only a few pairs of mutations are correlated. This includes a positive correlation of Q24K with L28M and L183P, a negative correlation of Q24K with V34I, and a positive correlation of V138L with V34I and negative correlation of V138L with L183P. We examined the order in which the strongly associated mutations may have occurred during the protein evolution. To this end, we reconstructed the phylogenetic tree of all GT-1a and GT-1b sequences. As expected, sequences correctly annotated with different genotypes are located on different branches of the tree. The mutation that served as the grounds for the separation of the sequences into atypical and typical, R30Q occurred independently in 45 internal nodes within the 1b subtree. We called these internal nodes as switch points. Some of the significantly associated mutations also occurred independently in several of these branches (Table S2). For each mutation that is overrepresented in the atypical set we also calculated its phylogeny-aware statistical significance by randomly sampling the same number of internal nodes at the same distance from the tip and then sampling the corresponding number of leaves in the induced subtrees. We performed 10,000 such samplings and in each case calculated the number of terminal nodes carrying the same mutation as the corresponding overrepresented mutation, and used this distribution to calculate the fraction of cases when the number of mutations the number of mutations in the random samples was larger than in the atypical set (Table 2). Only mutations Q24K, L28M, V34I, V138L, and L183P had a significant phylogeny-aware statistical significance. The mutation Q24K occurred in three branches independently: in a branch of closely related strains isolated in France in 2008 (marked with an asterisk in Figure 2), in several related sequences from Japan (marked with a dagger in Figure 2; detailed information on these two clades is presented in Figure S1), and in an isolated branch containing the single strain KC124940. Additionally, in four branches the mutation Q24R occurred. Arginine, as well as lysine, is positively charged, which emphasizes the importance of a compensating positive charge at this position. In all cases the parent residues in the switch point was glutamine, which suggests that the mutation Q24K/R is a compensating mutation for R30Q. L28M occurred in only one branch and prior to the switch point, and hence is probably unrelated to compensating R30Q. V34I occurred in seven branches and V34L in another three branches; in all 10 cases the residue in the switch point was a valine. Although all three residues at this position—valine, leucine, and isoleucine—are hydrophobic, valine is smaller than the other two, so maybe a larger residue is required at this position in the R30Q context. The mutation V34L was negatively correlated with Q24K/R. V138L occurred in six branches and V138I developed in additional four branches. In these 10 cases the parent residue at the switch point was a valine, again the change being from a smaller hydrophobic residue to a larger one. However, in another nine branches the parent residue at the switch point was already an isoleucine. In this case the trend was less pronounced: in the R30Q context a larger residue seemed to be preferred, but an isoleucine could be tolerated also in the wt. The mutation L183P happened only within one branch, and in the other five cases was present in the parent node at the switch point, so it does not appear to be compensating for R30Q. Additionally, this position can harbour a variety of hydrophobic and small amino acids. Sequence logos of GT-1b sequences with and without the R30Q mutation also demonstrate that these five positions demonstrate and amino acid profile that is closer to GT-1a than GT-1b.

Additionally, we have performed mutual information-based analysis for the atypical and typical sets of GT-1b sequences, as well as for GT-1a and GT-1b sequences with multi-Harmony and SDPfox in order to identify positions that discriminate between these groups (Table 3). Both methods identify, in addition to position 30, a number of positions that partially overlap with the results of our statistical analysis. However, these models are unaware of the underlying phylogenetic relationships between the viral strains, and thus cannot recapitulate the results of this study.

### 3.2. Analysis of Compensatory Mutations in GT1a-Failing Patients

To further study the importance of these mutations for the in vivo efficiency of DAAs, we examined sequences from 364 patients in the P.E.P.S.I. cohort. We detected eight GT-1a sequences carrying the RAM Q30R, where no clinical records about previous exposure to NS5A inhibitors were available for 7/8 sequences and 1/8 was DAA-naïve. In 2/8 samples we observed an additional K24Q mutation, and in one sample a K24N mutation. In position 28, for these patients we observe two mutations to a valine, and in one case to an alanine. Position 34 in all eight cases is occupied by an isoleucine, an amino acid that is consensus in the GT-1a, but also common in the GT-1b. Additionally, we observe one P183L mutations. These data may suggest that the resistance-associated variant Q30R can be compensated for by additional mutations. This fact should be taken into account when considering therapy options for patients with this variant.

We also analysed 11 additional viral samples from 7 P.E.P.S.I. patients who were infected with GT-1a viruses and who failed their NS5A inhibitor-containing treatment. The 5 baseline and 7 failure samples were sequenced using next generation sequencing to unveil minor strains within the viral quasispecies. None of the viruses carried NS5A RAMs at baseline. After treatment failure, 5/7 viruses contained variants carrying Q30R or Q30H with a frequency greater than 10%. 3/7 viruses displayed other RAMs relevant to their failure with a frequency greater than 10%: L31IML, H58HD, Y93YH, or Y83C. From the five failures with Q30RH with > 10% prevalence, no mutations in the residue 24 were found but minor variants with additional mutations in positions 28 and 34 were observed. I34V was observed in two cases (with frequencies greater than 1% and greater than 5%) both concomitant with the RAMs Q30R and Q30H. None of these I34V mutations were observed at baseline. Importantly, two or more mutations in these 11 sequences were observed in only 14% of other protein positions. M28I was observed with a frequency greater than 1% in one of the samples with Q30QRH. In this case we did not have access to the baseline sequence. Isoleucine does not occur at this position in GT-1b, but is also a large aliphatic amino acid, as the consensus leucine.

## 4. Discussion

In this study we describe epistatic interactions within the NS5A protein of HCV. We examined NS5A sequences with substitutions in the position 30 in GT-1b sequences from the HCV sequence database as well as in GT-1a clinically-derived samples from the P.E.P.S.I. Study. In GT-1a, the wt susceptible viruses carry glutamine in this position, while variants with the Q30R are associated with resistance to NS5A inhibitors. In GT-1b, the wt susceptible residue is an arginine. GT-1b variants displaying the R30Q substitution are susceptible to NS5A inhibitors and have been detected before treatment in 12% of Japanese patients [46].

Our analysis of 2777 GT-1b sequences confirmed that R30Q is a common polymorphism in the GT-1b, present in 5% of the sequences, and that this mutation occurred at 45 independent time points, as suggested by the reconstructed phylogenetic tree. R30Q has a negative effect on viral replication capacity (also known as viral fitness), with these viruses displaying a fitness of 44% compared to the wt [46]. Analysis of GT-1b sequences allowed us to identify mutations strongly associated with changes in the amino acid at position 30. Here we demonstrate that R30Q is strongly associated with the secondary mutations Q24K/R and V34L/I, which are, in turn, inversely correlated with each other. Interestingly, polymorphisms at positions 24 and 28 have been also reported at baseline in chronic patients from Japan [47,48].

Residues 24, 30 and 34 are located in the same region encompassing the flexible linker region and the amphipathic alpha-helix, *N*-terminal to the structured cytosolic portion of domain D1. This region has been suggested as the binding pocket for DAAs in different computational models [31,33,34,35,36,37]. However functional importance of individual residues in this region in the wt protein is not well understood. We note that Q24K/R restores the positive charge in this region that is lost upon the R30Q mutation, which may be important for some interactions of NS5A. According to the experimental NMR structures of the isolated amphipathic alpha-helix, residue 24 is located in a stable helical part of the amphipathic alpha-helix, whereas residue 30 is in its flexible end adjacent to the linker [29]. It must be noted that multiple models of the amphipathic helix, differing in their bending, have been suggested based on the NMR data (PDB IDs 1R7C through 1R7G, [29]). This diversity is the consequence of the different lipid composition of the membrane used in the experiments, or can be an artefact of the lack of the data on long-range constrains. However, in all the models, amino acid residues at positions 24 and 30 lie to one side of the helix that is opposite to its hydrophobic side. Thus, these residues either are in contact with the polar lipid heads, or are exposed to the cytosol. Moreover, in full-length model (discussed in detail below), position 30 can be located in the flexible linker outside the amphipathic helix [37].

Interestingly, residues 24, 30, and 34 all lie inside or close to a potential double SH3 domain-binding motif P^29^-xx-P-xx-P^35^. Although interaction with host factors containing SH3 domains has been reported for NS5A [49,50], there is no evidence yet that this particular motif takes part in such interactions. On the other hand, a P-xx-P-xx-P motif has been identified and structurally resolved in the sorting nexin 5 [51], where it plays a role in specific lipid binding. In NS5A, mutation P35A has been shown to slightly reduce virus infectivity, induce defects of virus assembly and not to have much effect on viral RNA replication [52]. Taken together, the existence of this conserved and potentially functional motif, as well as the presence of compensating mutations in and around it, suggest that these regions of NS5A plays a role in specific interactions with lipids or host factors.

Both Q24K/R and V34L/I mutations are likely to confer evolutionary advantage since they independently recur on different branches of the phylogenetic tree following the R30Q switch. Development of the mutation Q24K must not be the only way to restore NS5A function, since there are 104 strains with the R30Q mutation and no Q24K/R mutation our dataset. Of these 104 strains 65 carry the mutation V34L. The compensating action of V34L/I is more difficult to interpret. Valine, leucine and isoleucine are all aliphatic, but both leucine and isoleucine are larger than valine. Size could be the factor conferring the evolutionary advantage in the R30Q context. However, isoleucine can also be tolerated in the wt (R30) sequences. The mechanism of compensation of otherwise deleterious mutations is often such that first a mutation with no significant effect on fitness (“permissive”) is introduced at a different site of the protein sequence that epistatically interacts with the deleterious (resistance) mutation thus enabling the second change. This was observed, for example, in connection to the Oseltamivir resistance in influenza A H1N1 virus [53] and to protease inhibitor resistance in HIV-1 [54]. Thus, a larger hydrophobic amino acid at position 34 may be an example of such permissive mutations that can be tolerated in the sequence of a DAA-susceptible virus and facilitates future development of RAMs.

Additional and less strongly associated mutations to R30Q are V138L and L183P. We observe the mutation V138L several times independently and in one branch in combination with V34I. In all cases it happened after the R30Q mutation, as suggested by the reconstructed ancestral sequences. Additionally, several mutations V138I have been observed, also following the R30Q switch. Hence, the larger aliphatic amino acid residues are preferred in this position in combination with the R30Q mutation, as well as in position 34. The residue 138 is located in C-terminal part the structural cytosolic part of the domain D1, and from the available models it is unclear how it may interact with either residues 30 or 34, which are in the flexible linker between cytosolic subdomain of D1 and the amphipathic alpha-helix. However, this distal location may be an artefact of protein truncation required for in vitro expression and crystallization. Our data may indicate that in vivo this part of NS5A folds closer to the membrane and may take part in some interaction in coordination with residues 24, 30 or 34. All L183P mutations happen within one single branch, and in all five cases of the R30Q switch in it L183P was already present when the switch occurred, so it does not appear to be compensating for R30Q. This position can also harbour a variety of hydrophobic and small amino acids.

**Figure 3:**
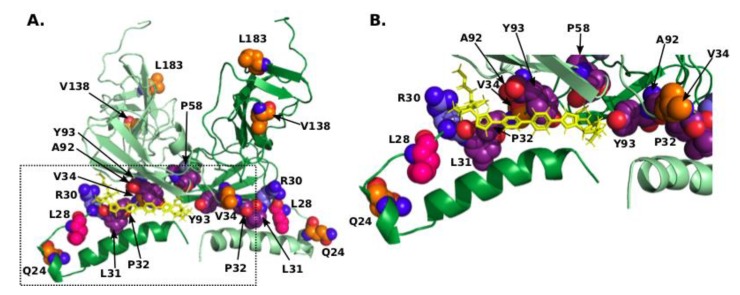
(**A**) Model of the dimeric complex comprising the amphipathic α-helix and cytosolic subdomain [37] with R30, compensating residues, and residues corresponding to RAMs shown in sphere mode with nitrogens coloured blue, oxygens coloured red, carbons of R30 coloured light-blue, of L28 coloured pink, of other compensating residues coloured orange, and of residues corresponding to RAMs coloured purple. Daclatasvir is shown as yellow sticks. The second chain of the dimer is shown in lighter colours. This model corresponds to mode-II in [37], which represents the potential high-affinity binding mode. (**B**) Close-up of the pocket occupied by Daclatasvir.

We have also studied the location of the identified correlated mutations in the context of one of the structural models of Daclatasvir binding suggested in the literature [37]. Unlike in other models, the inhibitor binds here in a non-symmetrical manner, but in concert with other models, it binds to the pocket between the amphipathic α-helix, cytosolic subdomain of D1 and the linker region between them (Figure 3). The two models proposed in this study correspond to two potential conformations of the membrane associated with NS5A: one in the endoplasmic reticulum membrane (mode-I) and the other in the lipid droplet membrane (mode-II). Based on these data from their modelling and experimental information, Nettles and colleagues [37] suggest that drug binding in mode-II irreversibly sequesters NS5A to lipid droplets and interferes with interactions of NS5A required to form replication complexes.

In this model, Daclatasvir interacts with amino acids in positions of RAMs 30, 31, 32, and 93. Interestingly, it also is in contact or in close proximity with several compensating positions identified in this study: the distance from Daclatasvir to L28 is 7.0 Å, and to V34 8.4 Å. Compensating residue 34 packs closely against residues corresponding to RAMs at positions 32, 92, and 93, and L28 is identified as a compensating residue and is also a known RAM (Figure 3). Additionally, residue 34 is located closely next to residue at position 161 from the opposite monomer (distance between carbon atoms of 3.6 Å indicates a hydrophobic contact), which also differs between GT-1a (F) and GT-1b (Y), and an invariant proline at position 163. Position 161, however, is not correlated with position 34 in our analysis. Residue 34, located at the end of the linker close to the structured cytosolic subdomain in this model, provides a hinge for the amphipathic helix/linker part of NS5A to move relative to this subdomain (Supplementary Movie S1), which may be important in the process of switch between the different oligomerization states. Mutation of this residue to a larger amino acid can shift the equilibrium between the states and displace the amphipathic helix.

The distance between residue Q24, the mutation of which is most strongly correlated with R30Q mutations, and Daclatasvir is 17.3 Å and the distance to R30 itself is 15.4 Å in this model, so the importance of this residue cannot be explained by direct or indirect interactions. It is located at the turn on the amphipathic helix and points away from the membrane, as well as the residue of R30 does. Since they are too far away to interact with each other, the above mentioned charge compensation here may indicate their joint involvement in some interaction, for example with a host factor, where charge plays a role. Residue 24 is located at the far end of the amphipathic helix, and its displacement between the states corresponding to mode-I and mode-II [37] is the largest (Supplementary Movie S1). Since mutation V34I anti-correlates with Q24K, and mutation of the hinge residue V34 can cause a large change in positioning of Q24, it is possible that either a change of position or a change of charge at amino acid 24 are needed to compensate the effect of the mutation at position 30, but not both.

Our data agree with the model of asymmetric binding of DAAs to NS5A suggested by Nettles et al. [37] and allow us to hypothesize about collective involvement of the compensating residues in specific interactions. In Barakat et al. [33] model of symmetric DAA binding with differently packed amphipathic helices, the compensating residues identified here seem to be located further from the drug-binding pocket, and do not make so many contacts with other residues of NS5A. However, they may interact with other unknown partners in this conformation as well.

Inhibitor binding is obviously not the biological role of the pocket formed by the amphipathic helix, linker region and cytosolic part of D1 in the virus replication cycle. Little is known about the endogenous interaction partners of this region. It has been, however, shown that a mutation at position W9 can abrogate the interaction with the host factor TIP47 [55], highlighting potential involvement of this region in protein-protein interactions with the host. Residue W9 lies next to residue V8, which was also identified as a candidate compensating residue (Table 2) but did not pass the significance threshold or receive enough phylogenetic support. V8 also lies only 10.7 Å away from the compensating residue V34 and may interact with it through a series of hydrophobic contact that include residues 7. Taken together, our data suggest that there are host factor interaction sites in this region yet to be discovered, and pinpointed here compensating residues may play a role in these interactions. Maintaining the charge balance between residues 24 and 30 described above may also be one of such factors. These data call for a large-scale study of epistatic interactions in NS5A that do not necessarily involve R30.

Mutations at the amino acid position 30 have strong relevance for GT-1a susceptibility to DAAs. Antiviral treatment constitutes an evolutionary pressure forcing viruses to alter their targets (in most cases viral proteins) through development of RAMs that modify the target and avoid the drug effect. Most RAMs, in spite of allowing the virus to replicate in the presence of drugs, hinder the protein function to some extent therefore decreasing the overall viral fitness [56]. That is the reason why resistance-associated-viruses (RAVs) are rare in therapy-naïve patients and in the absence of transmitted-resistance. In our seven failing patients from the P.E.P.S.I. cohort, the viruses succeeded to escape the treatment pressure through development of RAMs at position 30 and/or 93. Generation of RAMs in position 30, in turn, caused evolutionary pressure on other sites in epistatic interaction with it. Three out of five patients with the Q30RH RAM also developed mutations in positions 28 and 34. These compensatory mutations were detected in lower frequencies than the Q30RH, suggesting their subsequent development. Similar effect was showed with the ancestral state reconstruction for a maximum-likelihood phylogenetic tree comprising sequences from the HCV database, where I34V and M28I are predicted to occur after Q30RH. Thus, the observed in vivo mutation patterns concur with our hypothesis of positive epistasis in these pairs. However, the in vivo relevance of these associations for NS5A inhibitors resistance and therefore the utility of their inclusion in bioinformatics prediction systems like geno2pheno_[HCV]_ is still to be elucidated. We are aware that in this study the in vivo failure dataset is very limited, so additional longitudinal analysis is required to confirm whether I34V and M28I are always developed after Q30RH or whether the presence of baseline I34V and M28I may also contribute to the development of the Q30RH RAM. In addition, a possible role of compensatory mutations such as I34V and M28I in RAM persistence after the end of therapy should be addressed in future studies.

Epistatic interactions in the form of compensatory mutations are a known phenomenon accompanying antiviral, antibiotic, or antifungal therapy [57,58,59]. This phenomenon has been extensively described for human immunodeficiency virus 1 (HIV-1). Fitness decrease due to protease RAMs can be epistatically compensated by mutations in the target cleavage sites in the viral RNA as well as with secondary mutations in the protease itself [60,61,62]. The restoration effect of compensatory mutations may be so marked that RAMs may persist for long periods of time even in the absence of drug pressure, as shown for HIV [63], with direct but also indirect (persistence of RAVs increase resistance transmission) consequences for treatment success. In HCV, an epistatic effect similar to the one described here has been observed in NS3 protein [64], where Q80K mutation, which is also associated with reduced drug susceptibility, turns out to be a common polymorphism in GT-1a, and is also compensated for by secondary mutations that are structurally proximal. In contrast to R30Q in NS5A in GT-1b described here, Q80K occurred at a single point of the evolutionary history of the virus, and as well as for R30Q this point predated the introduction of DAAs. However, the HCV NS5A epistatic interactions described in this work require extensive future in vitro and in vivo analysis to confirm their relevance for patients’ treatment.

## 5. Conclusions

In conclusion, R30Q is a polymorphism in GT-1b (accounting for 5% of the sequences) that arises independently at different points during the virus’ evolution. It is significantly associated with secondary mutations Q24K/R and V34L/I in the same protein region, which partially restore its physicochemical properties and also arise independently multiple times. It is less significantly associated with mutations V138L and L183P. In vivo data also showed an association of the resistance-associated variant Q30R with mutations in the secondary positions described here. This might suggest that the identity of amino acids in these additional positions may have relevance for therapy selection.

## Figures and Tables

**Table 1:** Residues (one-letter code) associated with resistance to specific NS5A inhibitors.

Residue Number	Residue Identity in GT-1a	Residue Identity in GT-1b	Residue Identity in Experimentally Resolved Structures
28	M	L	M (1R7E)
30	Q	R	Q (1R7E)
31	L	L	L (1R7E)
32	P	P	L ^1^ (3FQM)
58	H	P	P (1ZH1, 3FQM)
92	A	A	A (1ZH1, 3FQM)
93	Y	Y	Y (1ZH1, 3FQM)

**^1^** Leucine at position 32 in the structure 3FQM was artificially introduced to enable protein purification. GT: sub/genotype for HCV.

**Table 2:** Statistical association of the selected positions with the atypical set. Mutations with significant *p*-values in Fisher’s exact test are in bold; mutations with significant support in the phylogenetic test are underlined.

Mutation	Atypical Set		Typical Set		*p*-value (Fisher’s Exact Test)	Phylogeny-Aware Statistical Significance
	# mutated aa	# WT aa	# mutated aa	# WT aa		
V8I	4	137	285	2351	3.977 × 10^−3^	0.962
Q24K	33	108	3	2633	1.754 × 10^−121^	0.000
L28M	5	136	13	2623	1.120 × 10^-4^	0.010
V34I	66	75	144	2492	7.108 × 10^−72^	0.000
K44R	9	132	377	2259	0.012	0.930
Q54H	13	128	850	1786	1.492 × 10^−8^	0.999
T83M	7	134	362	2274	4.221 × 10^−3^	0.886
S107T	1	140	128	2508	0.038	0.848
V121I	4	137	127	2509	0.380	0.595
T122R	3	138	42	2594	0.883	0.200
V138L	38	103	187	2449	1.455 × 10^−16^	0.002
V153L	10	131	385	2251	0.018	0.924
D171E	65	76	1219	1417	1.000	0.188
N180H	8	133	219	2417	0.340	0.448
L183P	60	81	426	2210	2.340 × 10^−15^	0.005

WT: wild type.

**Table 3:** Group-specific positions identified by mutual information-based tools. Positions that were also identified as correlated with the R30Q mutations in this study are shown in bold and underlined. Position 30 is shown in italics.

Tool	Atypical GT-1b vs. Typical GT-1b	GT-1a vs. GT-1b
Multi-Harmony	7, 8, 17, **24**, 25, 26, **34**, 37, 44, 48, 49, 52, 54, 55, 56, 62, 69, 73, 74, 75, 78, 83, 85, 101, 123, **138**, 164, 174, 181, **183**	7, 8, 14, 17, 21, **24**, 25, 26, **28**, *30*, **34**, 36, 37, 44, 46, 48, 54, 56, 58, 62, 64, 68, 71, 78, 81, 83, 85, 92, 93, 97, 101, 107, 108, 114, 116, 117, 121, 123, 124, 135, 137, **138**, 146, 153, 158, 161, 164, 166, 171, 174, 176, 180, 181, **183**, 222
SDPfox	**24**, *30*, 37, 62, 164, 174, 181	8, **24**, 25, *30*, 114

## Supplementary Materials

The following are available online at http://www.mdpi.com/2073-4425/9/7/343/s1, Supplementary File 1: Sequences used in this study, Supplementary File 2: Reconstructed phylogenetic tree with internal node numbers, Supplementary File 3: Reconstructed sequences in internal nodes. Supplementary Figure S1: Close-up representation of two densely populated branches containing the Q24K mutation. Supplementary Table S1: Statistical association of the selected positions with each other. Supplementary Table S2: Mutations in position with overrepresented 1a-specific amino acids in the switch points and the induced subtrees. Supplementary Movie 1: Morphing between two models.
