# Supplementary figures and images for: Epistatic Interactions in NS5A of Hepatitis C Virus Suggest Drug Resistance Mechanisms
Source: Genes (Basel). 2018 Jul 6;9(7):343. doi: 10.3390/genes9070343 (PMC6071292; doi:10.3390/genes9070343)

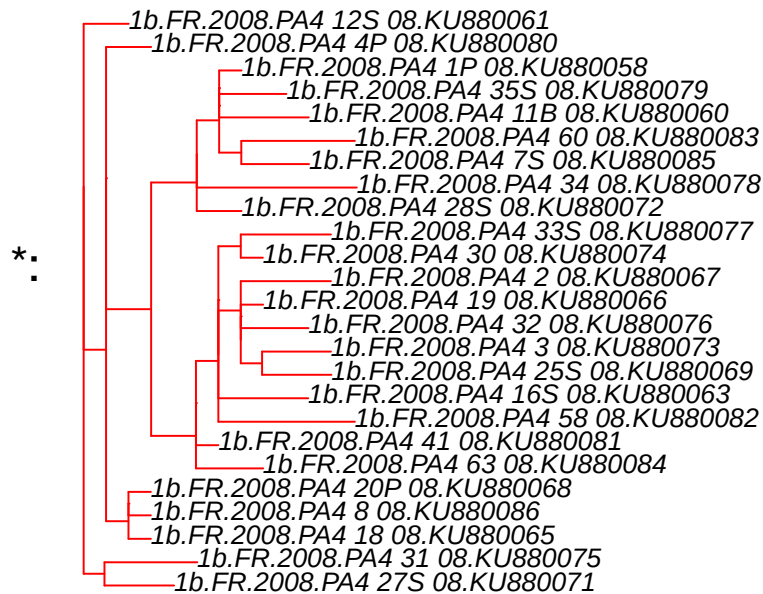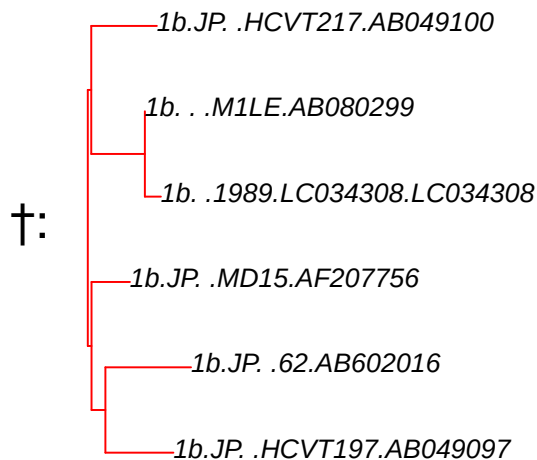

Supplement: Supplementary file 1 [file genes-09-00343-s001.zip › Suppl_Fig1_rev.pdf]
